# Evaluating youth-friendly health services: young people’s perspectives from a simulated client study in urban South Africa

**DOI:** 10.3402/gha.v8.26080

**Published:** 2015-01-23

**Authors:** Rebecca S. Geary, Emily L. Webb, Lynda Clarke, Shane A. Norris

**Affiliations:** 1 Department of Infection and Population Health, University College London, London, UK; 2 Department of Population Health, Faculty of Epidemiology and Population Health, London School of Hygiene and Tropical Medicine, London, UK; 3 Department of Infectious Disease Epidemiology, Faculty of Epidemiology and Population Health, London School of Hygiene and Tropical Medicine, London, UK; 4 MRC/Wits Developmental Pathways for Health Research Unit, Department of Pediatrics, Faculty of Health Sciences, University of the Witwatersrand, Johannesburg, South Africa

**Keywords:** adolescent health services, youth friendly, South Africa, simulated client

## Abstract

**Background:**

Few youth-friendly health services worldwide have been scaled up or evaluated from young people’s perspectives. South Africa’s Youth Friendly Services (YFS) programme is one of the few to have been scaled up. This study investigated young people’s experiences of using sexual and reproductive health services at clinics providing the YFS programme, compared to those that did not, using the simulated client method.

**Design:**

Fifteen primary healthcare clinics in Soweto were randomly sampled: seven provided the YFS programme. Simulated clients conducted 58 visits; young men requested information on condom reliability and young women on contraceptive methods. There were two outcome measures: a single measure of the overall clinic experience (clinic visit score) and whether or not simulated clients would recommend a clinic to their peers. The clinic visit score was based on variables relating to the simulated clients’ interactions with staff, details of their consultation, privacy, confidentiality, the healthcare workers’ characteristics, and the clinic environment. A larger score corresponds to a worse experience than a smaller one. Multilevel regression models and framework analysis were used to investigate young people’s experiences.

**Results:**

Health facilities providing the YFS programme did not deliver a more positive experience to young people than those not providing the programme (mean difference in clinic visit score: −0.18, 95% CI: −0.95, 0.60, *p*=0.656). They were also no more likely to be recommended by simulated clients to their peers (odds ratio: 0.48, 95% CI: 0.11, 2.10, *p*=0.331). More positive experiences were characterised by young people as those where healthcare workers were friendly, respectful, knew how to talk to young people, and appeared to value them seeking health information. Less positive experiences were characterised by having to show soiled sanitary products to obtain contraceptives, healthcare workers expressing negative opinions about young people seeking information, lack of privacy, and inadequate information.

**Conclusions:**

The provision and impact of the YFS programme are limited. Future research should explore implementation. Regular training and monitoring could enable healthcare workers to address young people’s needs.

Global health organisations, including the International Conference on Population and Development Plan of Action, the Maputo Plan of Action, and the World Health Organisation, (WHO) have called for the development of youth-friendly health services worldwide (1–5) . However, few such interventions have been scaled up or evaluated from young people’s perspectives (1–3) . The Youth Friendly Services (YFS) programme in South Africa is one of the few to have been scaled up to a national level. This programme (implemented in primary healthcare facilities) aims to improve the sexual and reproductive health of young men and women (6). The YFS programme has previously been highlighted as a successful model for implementing youth-friendly services within a public health system (7). However, evaluations of this programme have focussed on the attainment of pre-defined standards relating to the services provided, policies supporting adolescents’ rights and the clinic environment; just one study (conducted in 2005) investigated adolescents’ experiences (8–11) . The Department of Health (DoH) took over the management of this programme [previously known as the National Adolescent Friendly Clinic Initiative (NAFCI)] from the non-governmental organisation (NGO) loveLife in 2006, and no evaluations have taken place since (7, 12). Recent work has suggested that YFS provision is limited with just one of eight clinics in rural Mpumalanga reporting implementation of this programme (13).

This study had two objectives: the first was to investigate whether primary healthcare facilities providing the YFS programme delivered a more positive experience to young women requesting information on contraceptive methods and young men requesting information on condom usage than primary healthcare facilities not providing this programme. The second was to examine the characteristics of more and less positive experiences.

## Methods

The simulated client method, where the healthcare provider is not aware that a given client is participating in research, was used to address these objectives. This method removes observation bias and issues of privacy and confidentiality that may occur with direct observation or interviews with patients or healthcare workers. It has been used to study healthcare provider–client interactions by a number of studies in low- and middle-income countries (11, 14–20) .

Simulated clients have previously been recruited from groups including medical or university students, research assistants, nurses or people with particular socio-demographic characteristics (14, 18, 21). In this study, seven simulated clients (young men and women aged 22 years) were recruited from a long-running birth cohort of Soweto youth [described elsewhere (22, 23)] as it provided a practical sampling frame and these young people were within the target age group of the YFS programme. As members of the general population they were also likely to be more representative of the programme’s target population than research assistants. Simulated clients were randomly selected from a group of cohort participants who had previously participated in cohort health services-related research as this study formed an extension of that research programme. Young people were eligible to participate in the cohort health services-related research study if they tested positive for a sexually transmitted infection (STI) or urinary leukocytes at age 13 years. The simulated clients underwent training to present at clinics with two gender-specific scenarios, which were developed in collaboration with a clinician and two research nurses, with feedback from the simulated clients (Table 1).

**Table 1:** Summary of simulated client scenarios

Scenario	Details
Advice on condoms	The young man has heard that condoms can break and would like to know how reliable they are. The young man would also like a demonstration of how to put a condom on correctly. The young man is sexually active with a girlfriend. They use condoms (this information is only given if requested).
Advice on contraceptive methods	The young woman would like to know about how to prevent pregnancy. The young woman currently uses condoms (this information is only given if requested) but would like to learn about other methods. The young woman is sexually active with a boyfriend. They use condoms (this information is only given if requested).

A thorough response to either of these scenarios was defined as one which included: a discussion of correct condom use, the offer of a condom demonstration (to both young women and young men), assessment of STI exposure, STI/HIV counselling and testing, or referral. In addition, it would include the discussion of injectable contraceptives, oral contraceptives, and intra-uterine devices or systems with young women (24, 25).

Training involved conducting scenario role-plays with research nurses. Simulated clients were told that the aim of this study was to find out what young people’s experiences were like at the clinics, whether or not staff had been trained to try and make services friendly to young people. Local research assistants were present to provide language or cultural translations although this proved unnecessary.

Fifteen primary healthcare clinics, from a total of 29 in Soweto, were randomly sampled to receive 3–4 simulated client visits each. At least one male and one female simulated client visited each clinic between November 2011 and March 2012. Simulated clients were not asked to visit the clinics nearest their homes, or to visit a clinic more than once. Debrief questionnaires were conducted in English immediately following each visit.

## Statistical and qualitative analyses

### Definition of the exposure and outcome

The exposure (YFS provision) was defined based on DoH signs detailing the services provided at each clinic. It was not possible to blind simulated clients to which clinics provided the YFS programme given these signs. However, simulated clients were not told which clinics provided this programme, that these signs were present or that YFS provision was the focus of the study. No additional data were available or could be obtained on which facilities the DoH classified as implementing this programme. Other exposures of interest were clinic characteristics and simulated client socio-demographic characteristics. Clinic characteristics were collected using a short questionnaire administered to the nurse-in-charge at each clinic, who provided clinic-level informed consent. Socio-demographic characteristics of simulated clients were available having been collected previously as part of their participation in the Birth-to-Twenty cohort.

Because there were a large number of individual questions or potential outcomes, those which between them were felt to capture information that would define a good experience overall were combined into a single measure of the simulated clients’ overall experience during each clinic visit using principal components analysis. This clinic visit score was the first outcome measure. All collected variables were tabulated against each other, and those where there was evidence for correlation (p≤0.1) were included in the principal components analysis. Twenty-nine correlated variables were included in this measure, including those relating to the simulated clients’ interactions with staff, details of their consultation, privacy, confidentiality, the healthcare workers’ characteristics, and the clinic environment. A larger clinic visit score corresponds to a worse experience than a smaller score. As an illustration, the clinic visit with the smallest visit score (−4.71) had universally positive responses to the questions inputted into the principal components analysis. By contrast, the clinic visit with the largest visit score (2.47) had only three positive responses, which related to: the consultation room being clean and affording privacy, and the healthcare worker being respectful. The secondary outcome was whether or not simulated clients would recommend a clinic to their peers. Data were entered into Microsoft Access, statistical analyses were conducted in STATA 11, and framework analysis was performed in Microsoft Excel (26).

Multilevel regression models accounting for clustering at both simulated client and clinic levels were used to examine the association between YFS provision and the outcomes. Linear regression was used for clinic visit score, logistic regression for clinic recommendation. Univariable models were used to examine crude associations between YFS provision and each outcome, and between other exposures of interest (clinic size, local, or provincial government managing authority, presence of a peer educator, type of healthcare worker seen, simulated client gender, healthcare worker gender and age, maternal age at birth of the simulated client, and household SES) and outcomes. Any other exposure of interest associated with an outcome at *p*<0.05 in univariable models was included in the multivariable model for that outcome. Although experiences of health services may differ by gender, the small number of simulated clients (three males and four females) precluded conducting a quantitative analysis stratified by gender.

For the framework analysis, categorisation of themes was guided by Bruce’s Quality of Care for Family Planning framework (27). Additional themes could also be defined. Themes were compared between the consultations with the best clinic visit scores (n=15) and those with the worst score (n=15), and between male and female simulated clients. Data saturation, where no new or relevant information emerged, was reached.

Ethical approval was obtained from the London School of Hygiene and Tropical Medicine and The University of the Witwatersrand (Number: M110360). Permission to work with the clinics was granted by the relevant provincial and district health authorities and informed consent was obtained from all simulated clients and the nurse-in-charge at each clinic.

## Results

Fifty-eight clinic visits were conducted, 30 by female simulated clients and 28 by males. Thirteen clinics received four visits, two from male simulated clients and two from females: two clinics received three visits. Data from two of the 58 visits were not included in the analyses because the simulated client revealed their participation in the study. Refresher training was provided to these simulated clients.

Table 2 presents the clinic and simulated client characteristics. Of the 15 clinics sampled, eight were small (<6 nurses; 53%) and run by the local health authority. Approximately half of clinics provided the YFS programme (n=7) of which five were small, local authority clinics.

**Table 2:** Clinic and simulated client characteristics

Clinic characteristics (N=15)	% (N)
YFS provided	
Yes	46.67 (7)
No	53.33 (8)
Clinic size	
Small (<6 nurses)	53.33 (8)
Large (>10 nurses)	46.67 (7)
Clinic authority	
Local	53.33 (8)
Provincial	46.67 (7)
groundBREAKER peer educator	
Yes	26.67 (4)
No	73.33 (11)
Simulated client characteristics (N=7)	
Simulated client gender	
Female	57.00 (4)
Male	42.00 (3)
Population group	
Black	100.00 (7)
Maternal education at birth	
Secondary	100.00 (7)
Maternal age at birth	
<19	14.29 (1)
20–24	28.57 (2)
25–29	28.57 (2)
30–34	14.29 (1)
≥35	14.29 (1)
Household SES	
1	0.00 (0)
2	28.57 (2)
3	28.57 (2)
4	0.00 (0)
5	28.57 (2)
Missing	14.29 (1)

SES=Socio-economic status, derived by principal components analysis of household assets (electricity, television, car, fridge, washing machine, and telephone) collected from caregivers at enrolment into the cohort.

Table 3 illustrates some of the characteristics of the clinic visits; >70% (n=43) of consultations were with a nurse or sister and >85% were with a female healthcare worker (n=49). Only one variable was statistically significantly associated with YFS provision: whether a simulated client felt that the healthcare worker seemed happy to talk about condoms or other contraceptive methods with them. However, this association was driven by 14% of consultations at non-YFS clinics having *no discussion* of condoms (males) or other contraceptive methods (females) and a similar proportion of consultations at YFS clinics involving a healthcare worker who *did not seem happy to talk about condoms or other contraceptive methods* with them. Since these two situations are quite similar, the observed association is unlikely to be meaningful.

**Table 3:** Characteristics of the clinic visits (N=56)

	YFS not provided % (N)	YFS provided % (N)	All clinics % (N)	*p* a
Clinic visit level variables (N=56)				
Healthcare worker seemed happy to talk about contraceptives or condoms				
No	0.00 (0)	14.81 (4)	7.14 (4)	
No discussion	13.79 (4)	0.00 (0)	7.14 (4)	0.013^b^
Yes	86.21 (25)	85.19 (23)	85.71 (48)	
Simulated client felt respected by the healthcare worker				
No	3.45 (1)	11.11 (3)	7.14 (4)	0.343
Yes	96.55 (28)	88.89 (24)	92.86 (52)	
Simulated client perceived the clinic to have convenient opening hours				
No	13.79 (4)	11.11 (3)	12.50 (7)	1.000
Yes	86.21 (25)	88.89 (24)	87.50 (49)	
Simulated client was told consultation would be confidential				
No	65.52 (19)	62.96 (17)	64.29 (36)	0.842
Yes	34.48 (10)	37.04 (10)	35.71 (20)	
Simulated client felt consultation would be confidential				
No	20.69 (6)	22.22 (6)	21.43 (12)	0.889
Yes	79.31 (23)	77.78 (21)	78.57 (44)	
Simulated client felt that the consultation area afforded privacy				
No	24.14 (7)	18.52 (5)	21.43 (12)	0.609
Yes	75.86 (22)	81.48 (22)	78.57 (44)	
Consultation was interrupted				
Yes	13.79 (4)	33.33 (9)	23.21 (13)	0.116
No	86.21 (25)	66.67 (18)	76.79 (43)	
Number of interruptions				
0	86.21 (25)	66.67 (18)	76.79 (43)	
1	6.90 (2)	22.22 (6)	14.29 (8)	
2	0.00 (0)	7.41 (2)	3.57 (2)	0.125
3	3.45 (1)	3.70 (1)	3.57 (2)	
4	3.45 (1)	0.00 (0)	1.79 (1)	
Simulated client felt that the healthcare worker gave them their full attention				
No	17.24 (5)	14.81 (4)	16.07 (9)	1.000
Yes	82.76 (24)	85.19 (23)	83.93 (47)	
Simulated client felt that the healthcare worker was interested in their questions				
No	24.14 (7)	18.52 (5)	21.43 (12)	0.609
Yes	75.86 (22)	81.48 (22)	78.57 (44)	
The healthcare worker gave advice or condoms				
No	13.79 (4)	3.70 (1)	8.93 (5)	0.353
Yes	86.21 (25)	96.30 (26)	91.07 (51)	
Simulated client felt comfortable talking to the healthcare worker about contraceptives, or felt comfortable during the condom demonstration				
No	6.90 (2)	3.70 (1)	5.36 (3)	
No demonstration or discussion	13.79 (4)	11.11 (3)	12.50 (7)	1.000
Yes	79.31 (23)	85.19 (23)	82.14 (46)	
Simulated client felt able to ask all the questions they had				
No	31.03 (9)	22.22 (6)	26.79 (15)	0.457
Yes	68.97 (20)	77.78 (21)	73.21 (41)	
Healthcare worker answered all the questions the simulated client asked				
No	17.24 (5)	7.41 (2)	12.50 (7)	0.424
Yes	82.76 (24)	92.59 (25)	87.50 (49)	
Simulated client rating of the clinic visit experience				
Excellent	51.72 (15)	66.67 (18)	58.93 (33)	
Good but with room for improvement	24.14 (7)	22.22 (6)	23.21 (13)	
Neither good nor bad	6.90 (2)	3.70 (1)	5.36 (3)	0.723
Unsatisfactory	10.34 (3)	7.41 (2)	8.93 (5)	
Very unsatisfactory	6.90 (2)	0.00 (0)	3.57 (2)	

a Where any cell values are less than five, *p*-values are from Fisher’s exact test. Where cell values are five or more, *p*-values are from Pearson’s chi-squared test.

b Statistically significantly associated with the provision of the YFS programme (*p*<0.05).

There were also no statistically significant differences observed between clinics that provided the YFS programme and those that did not in univariable analyses in terms of clinic size, managing authority, presence of a peer educator, healthcare worker gender, healthcare worker age and level, cleanliness, waiting times, whether the clinic was perceived to be welcoming, and the provision of information and education materials (data not shown). Sexual health histories were not taken in any consultation, nor was HIV/STI testing offered. Counselling on HIV/STI prevention was rarely provided, and correct condom usage was demonstrated in only half (59%) of consultations with males and to no female simulated clients.

There was no evidence that clinics that provided the YFS programme delivered a more positive experience to simulated clients than clinics that did not provide this programme, adjusting for the effect of simulated client gender, healthcare worker age, and clustering (mean difference in clinic visit score: −0.18, 95% CI: −0.95, 0.60, *p*=0.656) (Table 4). There was strong evidence that male simulated clients had generally more positive experiences than female simulated clients, with a mean difference of 1.52 in clinic visit score between consultations conducted by males and females (*p*=0.009). There was also some evidence that consultations with older (compared to younger) healthcare workers were more positive experiences, adjusting for the effect of the provision of the YFS programme, simulated client gender and clustering (*p*=0.041). For each one-unit increase in healthcare worker age group, the mean difference in clinic visit score was −0.59. There was no evidence that clinics that provided the YFS programme were more likely to be recommended by simulated clients to their peers than those that did not, adjusting for the effect of maternal age at birth, healthcare worker age and clustering at the simulated client and clinic level (odds ratio: 0.48, 95% CI: 0.11, 2.10, *p*=0.331) (Table 5).

**Table 4:** Crude and adjusted multilevel models of the association between the provision of the YFS programme and clinic visit score

	Clinic visit score			
	
	Mean (SD)	Regression coefficient	95% CI	*p*
Univariate multilevel model				
YFS provided				
No	0.06 (1.47)	Reference		
Yes	−0.13 (1.82)	−0.12	−0.86, 0.62	0.748
Multivariable multilevel model				
YFS provided				
No	0.06 (1.47)	Reference		
Yes	−0.13 (1.82)	−0.18	−0.95, 0.60	0.656
Simulated client gender				
Female	0.81 (1.06)	Reference		
Male	−0.93 (1.68)	−1.52	−2.65, −0.38	0.009
Healthcare worker age				
20–29	1.38 (0.67)			
30–39	0.02 (1.37)	−0.59^a^	−1.15, −0.02	0.041
40+	−0.72 (2.01)			

For ordered categorical exposures, where tests for trend performed better than categorical tests in univariate, multilevel models, the variable was fitted as a continuous variable for use in multivariable, multilevel models.

**Table 5:** Crude and adjusted multilevel models of the association between the provision of the YFS programme and whether a simulated client would recommend a clinic

	Would recommend clinic			
	
	% (N)	Odds ratio	95% CI	*p*
Univariate multilevel model				
YFS provided				
No	86.21 (25)	1 (Reference)		
Yes	74.07 (20)	0.46	0.12, 1.78	0.260
Multivariable multilevel model				
YFS provided				
No	86.21 (25)	1 (Reference)		
Yes	74.07 (20)	0.48	0.11, 2.10	0.331
Maternal age at birth				
≤19	100.00 (4)			
20–24	87.50 (14)	0.57	0.30, 1.06	0.076
25–29	85.71 (18)			
30–34	60.00 (3)			
≥35	60.00 (6)			
Healthcare worker age				
20–29	57.14 (4)			
30–39	78.12 (25)	2.49	0.81, 7.66	0.113
40+	94.12 (16)			

### Characteristics of more and less positive clinic experiences

Less positive consultations were those where information was not given, privacy was lacking, and simulated clients experienced unnecessary barriers or negative opinions about seeking information. More simulated clients at more positive consultations reported that the healthcare worker was friendly, knew how to talk to young people, treated them with respect, and appeared to value them seeking information.

A common unnecessary barrier was that in the majority of consultations, female simulated clients were told that they would be required to return and show soiled sanitary products before they would be prescribed contraceptives. However, South African guidelines state that the initiation of hormonal contraceptives should not be restricted to menstruation (24). One female simulated client said: ‘She said I must come immediately I have my period and that I will have to show them the pad as many girls are coming when they are not on their period and are lying’ [Simulated Client 5 (female), Clinic 12 (not YFS)].

Simulated clients perceived that the healthcare worker valued them seeking information at the majority of the more positive consultations, but not at the majority of less positive consultations. One male simulated client reported that; ‘When I said I wanted to ask about condoms she said that she doesn’t have time because she has to see those young girls for injection. Then she just called to the other lady (patient) to come in so she can do pills and injections’ [Simulated Client 2 (male), Clinic 12 (not YFS)]. In addition, in the less positive consultations, information was sometimes not given at all, or it was medically inaccurate.

Both female and male simulated clients, particularly those who had children, reported that healthcare workers expressed surprise at them seeking information on contraceptives or condoms. One female simulated client described the healthcare worker’s reaction: ‘She said that I have a baby now, I should know better than getting information on prevention from here’ [Simulated Client 5, Clinic 3 (YFS)].

Judgmental attitudes were often linked to healthcare workers not providing certain information. For example, one female simulated client said: ‘When I came in she just stared at me. I said I wanted to ask about the different methods of prevention and before she answered she asked how old my baby is. When I was surprised and asked how she could know I had a child she said she could see it in my body. She said I should take the injection and that we shouldn’t go into the other methods. When I asked why she recommends the injection she asked how old I am and said that they don’t recommend pills for young people because they are careless. I said I am not a party person’ [Simulated Client 5 (female), Clinic 3 (YFS)]. Despite this simulated client’s assurances the healthcare worker refused to give information on user-controlled methods.

### Characteristics of more and less positive clinic experiences by gender

More male than female simulated clients reported that the healthcare worker they consulted knew how to talk to young people and treated them respectfully. This may reflect different experiences by males and females, different expectations or both. However, judgmental attitudes were more commonly exhibited towards female than male simulated clients. Male and female simulated clients emphasised healthcare worker behaviour and attitudes as the most important factors in determining more and less positive experiences.

## Discussion

There was no evidence that clinics providing the YFS programme provided a more positive experience to simulated clients, or were more likely to be recommended by simulated clients to their peers, than those not providing this programme. These results are consistent with those of an earlier study where clinics providing NAFCI were no more likely than facilities not providing NAFCI to provide a more positive experience to young simulated clients seeking HIV tests (11).

Positive and negative experiences were predominantly determined by the healthcare worker’s attitudes and behaviour. This is in line with the findings of other studies that the characteristics most valued by young people are staff attitudes and confidentiality, and that improving services for young people should focus on changing attitudes rather than addressing structural issues (28–30) . However, these findings offer important insight because the YFS programme is one of the few such interventions to have been scaled up to a national level. Although the YFS programme includes healthcare worker training, these results indicate a need for improvements in healthcare workers’ capacity to deliver positive experiences to young people, to address young people’s needs for information, contraceptive methods, and testing (for HIV/STIs and pregnancy), and to maintain confidentiality.

The limited discussion of some hormonal contraceptive methods and of condoms and the absence of condom demonstrations to young women or offers of HIV/STI tests are a cause for concern, particularly in a country with a high prevalence of HIV and unwanted pregnancy among young people (31, 32). Positive engagement with effective sexual and reproductive health services in early adolescence could help reduce incidence of HIV and adolescent pregnancy (33–35) . However, findings from rural South Africa suggest that healthcare workers may not always agree to see young adolescents alone (although the legal age at which young people can access health services independently is 12 years), or may breach confidentiality to their parents, which should be addressed through training (13).

## Strengths and limitations

A limitation of this study related to the definition of the exposure: YFS provision. At six of the seven sampled clinics identified as providing the YFS programme by the DoH, the nurse-in-charge reported that the clinic had not been involved with either NAFCI or YFS; lack of implementation of the YFS programme therefore seems to be a likely explanation for the lack of evidence for an association between the provision of the YFS programme and young people’s experiences. However, this does not mean that this programme would prove effective, even with adequate implementation. None of the nurses in charge at the eight clinics that were not identified by the DoH as providing the YFS programme reported that the clinic provided this programme. In the absence of process evaluation data to define YFS implementation, a pragmatic decision was taken to use the available DoH information to define YFS provision. Further research, including process evaluations, could establish whether the lack of observed impact indicates that the YFS programme is an ineffective intervention, or an intervention that has not been well implemented (36).

Another limitation was that simulated clients were all the same age, towards the older end of the YFS programme’s target age group and no simulated clients lived in households in the poorest household SES quintile. However, ethical approval was not granted to recruit adolescents aged less than 18 years of age. Therefore, these results may not be generalisable to younger adolescents or those living in the poorest households. Other simulated client studies have reported that simulated clients from poorer households were more reluctant to initially visit, or return to health facilities than those from wealthier households due to expectations of and previous experiences of negative treatment [89]. Including the experiences of young men, who are often missed from research on young people’s sexual and reproductive health, was a strength of this study, as was the use of the simulated client method to capture the realities of young women’s and young men’s experiences (37). As participants in a cohort study, these simulated clients have had repeated interactions with research nurses. They may therefore have been more confident when interacting with healthcare workers and have had more positive experiences than other young people. However, a wide range of experiences were described, the characteristics of more and less positive experiences are in line with other studies, and data saturation was reached, lending confidence in these results.

The South African DoH has acknowledged the limited success of the YFS programme (38). With improved implementation, this programme could increase the capacity of healthcare workers to provide positive experiences and high quality services to young people. These findings suggest five key changes that could enhance the implementation of youth-friendly health services and improve young people’s experiences of requesting information on condoms and contraceptive methods. These changes could be implemented through a dedicated programme such as the YFS programme, or through broader health systems channels.

1. Training for all healthcare workers should emphasise the need to provide non-judgmental and confidential services for young people.
2. The information given to young people should be comprehensive; young women requesting information on contraceptive methods should be informed of all the available options. Discussion and provision of contraceptive methods should not be limited based on judgmental attitudes about young people’s behaviour.
3. Sexual histories should be taken where time allows.
4. Condom demonstrations and HIV/STI tests should be offered to all young people requesting information or other services related to condoms or contraceptive methods.
5. Healthcare workers’ performance should be monitored regularly where possible and feedback and additional training provided as required and on request.

These changes could increase young people’s utilisation of health services and promote better health outcomes.

## Implications and contribution

Few studies have evaluated youth-friendly services from young people’s perspectives. YFS provision was not associated with more positive experiences; however, this may reflect limited implementation. Regular training and monitoring could enable healthcare workers to address young people’s needs for information and services in a non-judgmental way, and maintaining confidentiality. This may facilitate increases in young people’s utilisation of health services and promote better health outcomes.

## References

[1] World Health Organisation (2002). Adolescent friendly health services: an agenda for change.

[2] UNFPA (1995). International conference on population and development (ICPD) (Cairo 1994) – programme of action.

[3] The African Union Commission (2006). Universal access to comprehensive sexual and reproductive health services in Africa: Maputo Plan of Action for the operationalization of the continental policy framework for sexual and reproductive health rights 2007–2010.

[4] Resnick MD, Catalano RF, Sawyer SM, Viner R, Patton GC (2012). Seizing the opportunities of adolescent health. Lancet.

[5] UNAIDS (2002). Summary of the declaration of commitment on HIV/AIDS.

[6] Dickson-Tetteh K, Pettifor A, Moleko W (2001). Working with public-sector clinics to provide adolescent-friendly services in South Africa. Reprod Health Matters.

[7] Ashton J, Dickson K, Pleaner M (2009). The evolution of the national adolescent friendly clinic initiative in South Africa.

[8] Dickson KE, Ashton J, Smith J-M (2007). Does setting adolescent-friendly standards improve the quality of care in clinics? Evidence from South Africa. Int J Qual Health Care.

[9] loveLife (2004). 2004 report on activities and progress.

[10] loveLife (2007). HIV prevention in the Melmoth area, KwaZulu-Natal: an assessment of effects associated with loveLife’s intervention.

[11] Matthews C, Guttmacher S, Fisher A, Mtshizana Y, Nelson T, McCarthy J (2009). The quality of HIV testing services for adolescents in Cape Town, South Africa: do adolescent-friendly services make a difference?. J Adolesc Health.

[12] loveLife (2008).

[13] Geary RS, Gómez-Olivé FX, Kahn K, Tollman S, Norris SA (2014). Provision of youth friendly services in rural South Africa. BMC Health Serv Res.

[14] Schuler SR, McIntosh EN, Goldstein MC, Pande BR (1985). Barriers to effective family planning in Nepal. Stud Fam Plann.

[15] Larke N, Cleophas-Mazige B, Plummer ML, Obasi AIN, Rwakatare M, Todd J (2010). Impact of the MEMA kwa Vijana adolescent sexual and reproductive health interventions on use of health services by young people in rural Tanzania: results of a cluster randomized trial. J Adolesc Health.

[16] Nalwadda G, Tunmwesigye NM, Faxelid E, Byamugisha J, Mirembe F (2011). Quality of care in contraceptive services provided to young people in two Ugandan districts: a simulated client study. PLoS One.

[17] Renju J, Andrew B, Kishamawe C, Kato C, Changalucha J, Obasi A (2010). A process evaluation of the scale up of a youth friendly health services initiative in northern Tanzania. J Int AIDS Soc.

[18] Madden JM, Quick JD, Ross-Degnan D, Kalfe KK (1997). Undercover careseekers: simulated clients in the study of health provider behaviour in developing countries. Soc Sci Med.

[19] Rowe AK, Onikpo F, Lama M, Deming MS (2012). Evaluating health worker performance in Benin using the simulated client method with real children. Implement Sci.

[20] Davey-Smith G, Mertens T (2004). What’s said and what’s done: the reality of sexually transmitted disease consultations. Public Health.

[21] Huntington D, Schuler SR (1993). The simulated client method: evaluating client-provider interactions in family planning clinics. Stud Fam Plann.

[22] Richter L, Norris S, de Wet T (2004). Transition from birth to ten to twenty: the South African cohort reaches 13 years of age. Paediatr Perinat Epidemiol.

[23] Richter L, Norris S, Pettifor J, Yach D, Cameron N (2007). Cohort Profile: Mandela’s children: the 1990 birth to twenty study in South Africa. Int J Epidemiol.

[24] Department of Health Republic of South Africa (2012). National contraception and fertility planning policy and service delivery guidelines.

[25] Department of Health Republic of South Africa (2012). National contraception clinical guidelines.

[26] STATA Corp (2011). STATA 11. College Station.

[27] Bruce J (1990). Elements of the quality of care: a simple framework. Stud Fam Plann.

[28] Langhaug LF, Cowan FM, Nyamurera T, Power RM, The Regai Dzive Shiri Study Group (2003). Improving young people’s access to reproductive health care in rural Zimbabwe. AIDS Care.

[29] Erulkar AS, Onoka CJ, Phiri A (2005). What is youth-friendly? Adolescents’ preferences for reproductive health services in Kenya and Zimbabwe. Afr J Reprod Health.

[30] World Health Organisation (2001). Global consultation on adolescent friendly health services. A consensus statement.

[31] Panday S, Makiwane M, Ranchod C, Letsoalo T (2009). Teenage pregnancy in South Africa: with a specific focus on school-going learners.

[32] UNAIDS (2012).

[33] Government of the Republic of South Africa Children’s Act.

[34] Shisana O, Rehle T, Simbayi LC, Zuma K, Jooste S, Pillay-van-Wyk V (2009). South African national HIV prevalence, incidence, behaviour and communication survey 2008: a turning tide among teenagers?.

[35] Tylee A, Haller DM, Graham T, Churchill R, Sanci LA (2007). Adolescent Health 6: youth-friendly primary-care services: how are we doing and what more needs to be done?. Lancet.

[36] Rychetnik L, Frommer M, Hawe P, Shiell A (2002). Criteria for evaluating evidence on public health interventions. J Epidemiol Community Health.

[37] Saewyc EM (2012). What about the boys? The importance of including boys and young men in sexual and reproductive health research. J Adolesc Health.

[38] Department of Health Republic of South Africa (2012). National adolescent and youth friendly health services strategy 2012.

